# Supplementary material for: Cell volume regulates terminal differentiation of cultured human epidermal keratinocytes
Source: J Cell Sci. 2025 Sep 8;138(17):jcs264242. doi: 10.1242/jcs.264242 (PMC12450470; doi:10.1242/jcs.264242)
Supplement: Supplementary information [file joces-138-264242-s1.pdf]

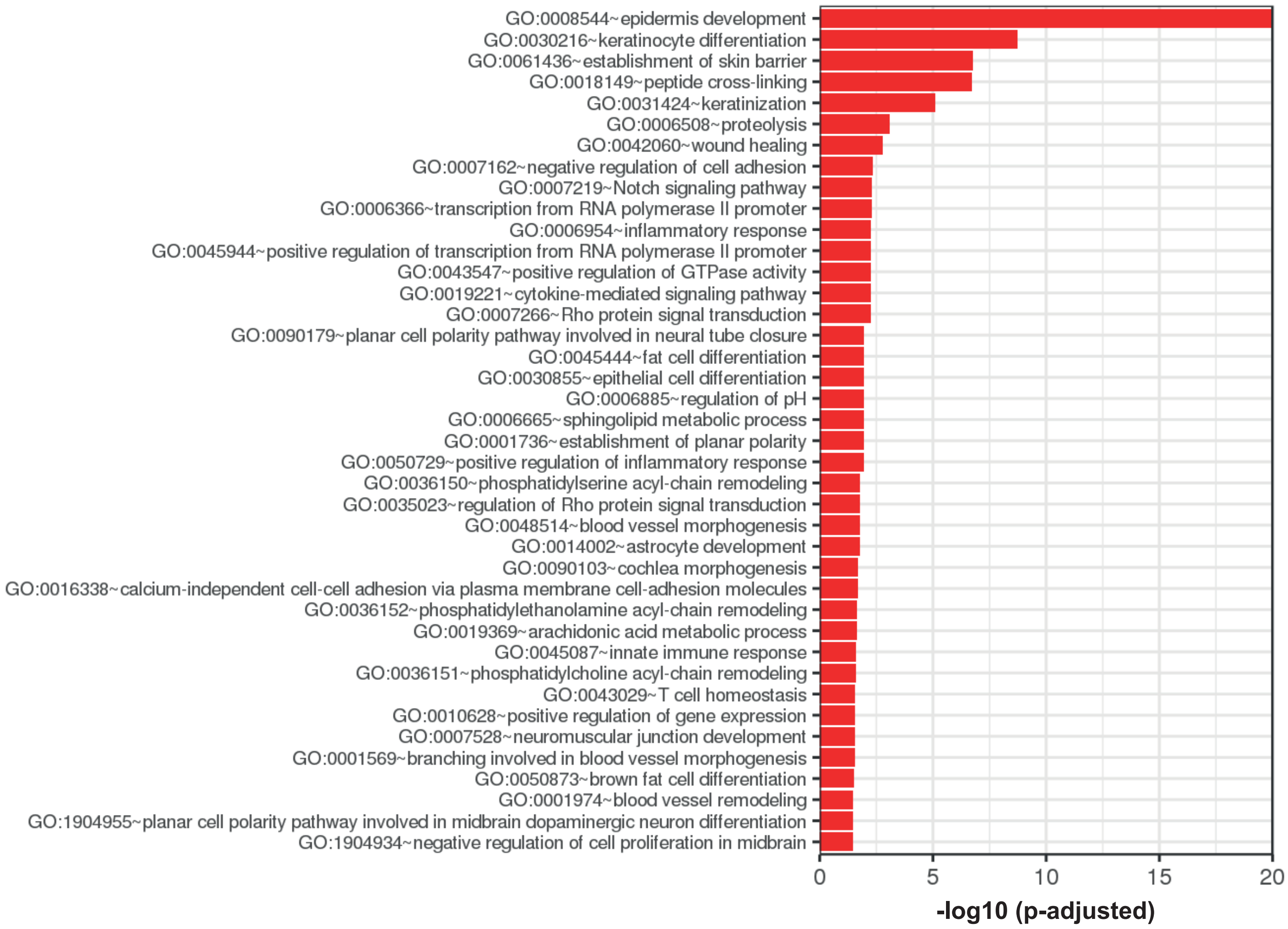

Fig. S1. Gene ontology (GO) analysis of differentially expressed genes. Gene ontology (GO) analysis of differentially expressed genes expressed between S1 and S2 at 12h (LFC > 1 or LFC < -1, padj <0.05). Differentially expressed genes were clustered and tested for GO enrichment using GeneSCF v1.1-p2 (Subhash & Kanduri, 2016). GO term enrichment was statistically tested (Fisher's exact test) and significant p-values were adjusted for multiple testing (p-adjusted). The top 40 most significantly enriched GO terms (p-adjusted < 0.05) are shown. GO terms (y-axis) are plotted against the -log10 of their adjusted p-value (p-adjusted). Figure is reproduced from the GENEWIZ Differential Gene Expression Analysis Report.

Journal of Cell Science • Supplementary information

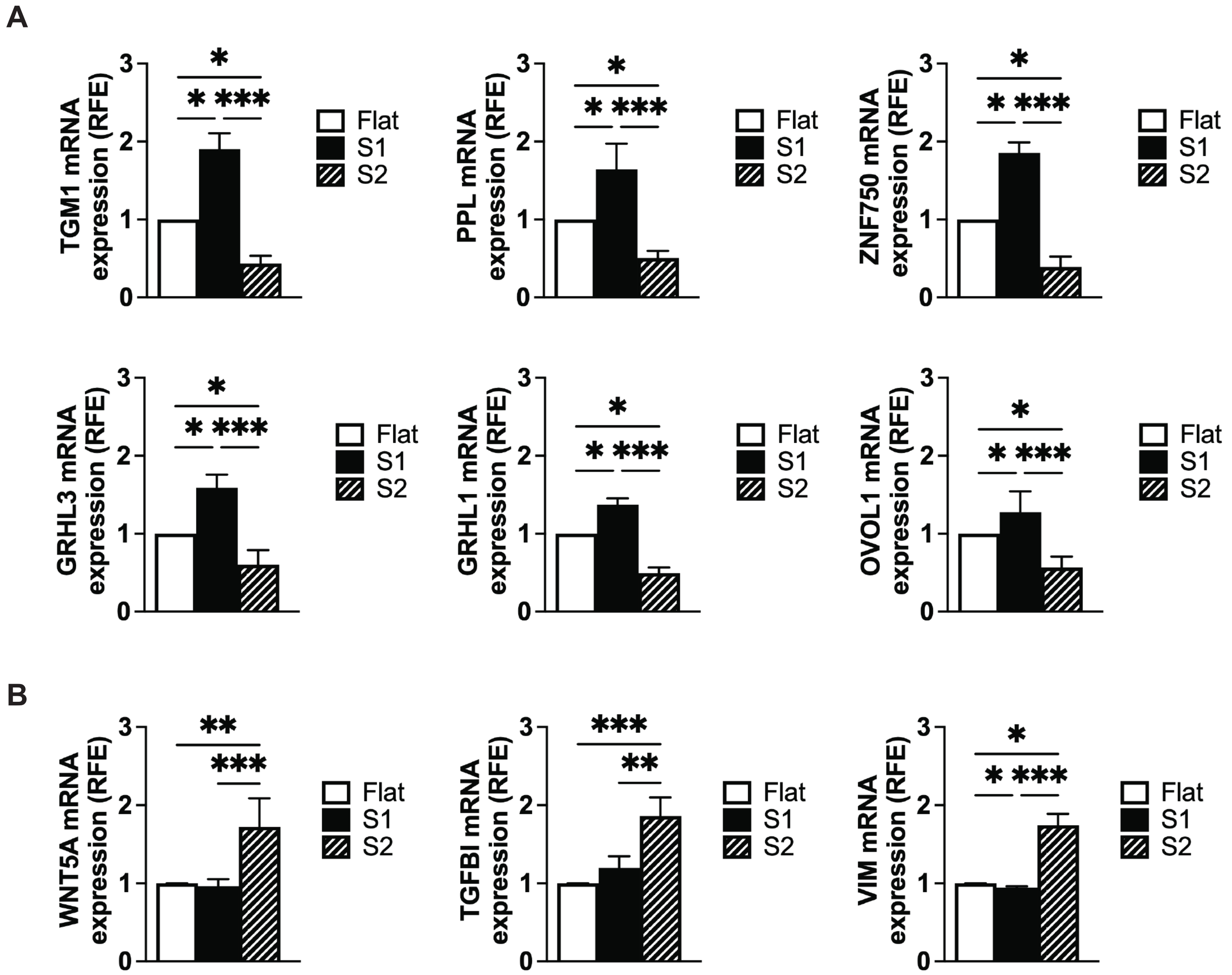

Fig. S2. Real-time quantitative PCR of differentially expressed genes. Quantification of messenger RNA (RT-qPCR) expression of genes identified by bulk RNA sequencing as being differentially upregulated on S1 (A) or S2 (B) substrates. Cells were cultured on flat, S1 or S2 substrates for 12h. Results are from three independent experiments with 4 technical replicates per substrate. Statistics: Kruskal-Wallis (+ Dunn's test). RFE: relative fold expression (compared to flat substrates). * p-value < 0.05, ** p-value < 0.01, *** p-value < 0.001.

Journal of Cell Science • Supplementary information

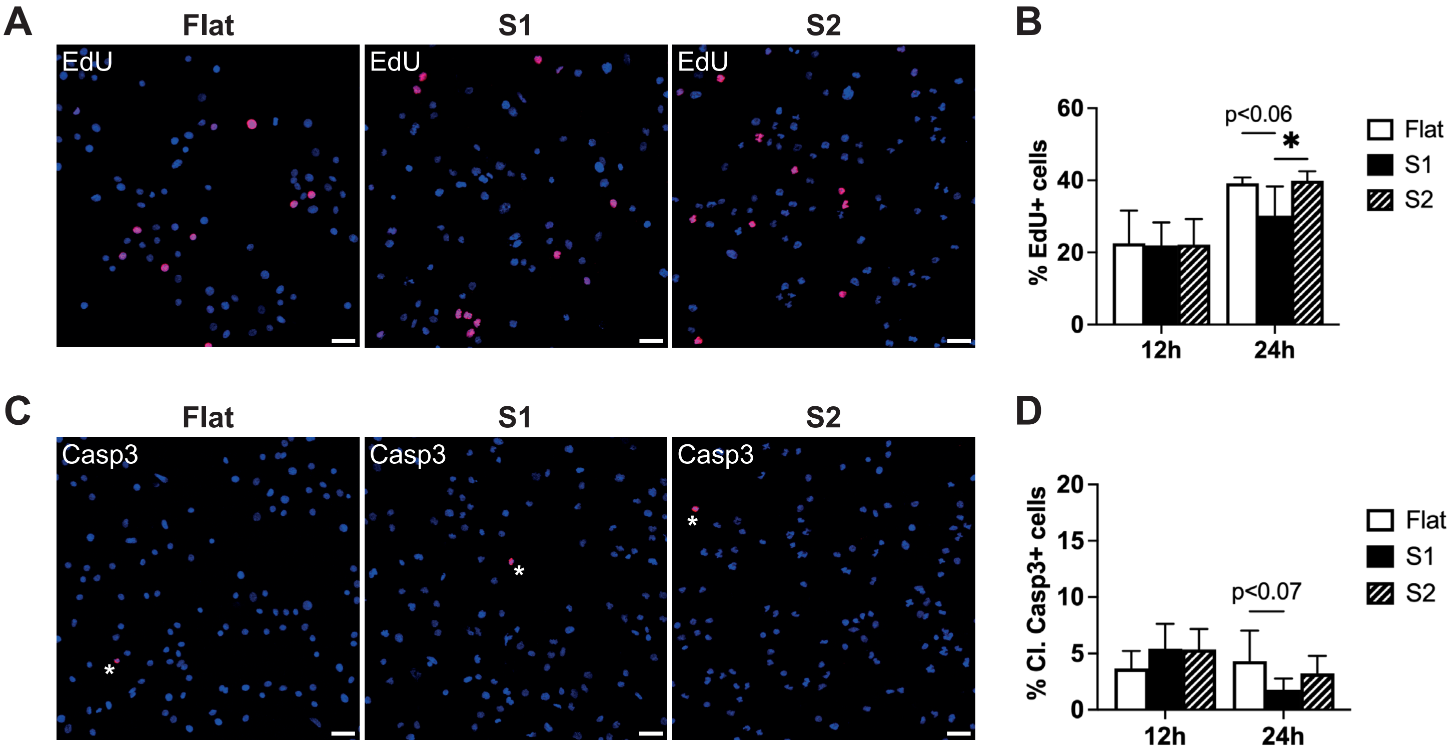

Fig. S3. Proliferation and apoptosis on different substrates (A, B) Representative images (A) and quantification (B) of 5-ethynyl-2'-deoxyuridine (EdU, red) incorporation by cells cultured on flat, S1 and S2 substrates for 12h (A, B) or 24h (B). DAPI (blue): nuclei. (C, D) Representative images (12h; C) and quantitation (12h, 24h; D) of cleaved caspase-3 antibody labelling (Casp3, red with blue DAPI counterstain). Asterisks in (C) represent rare apoptotic (Casp3+) cells. (B, D) Quantification based on >1000 cells per experiment. Results are from three independent experiments performed with three technical replicates per substrate. Scale bars: 50 µm. Statistics: two-way ANOVA (incl. Tukey's) test. Only (near-) significant p-values are shown. Error bars: mean + SD. * p-value < 0.05.

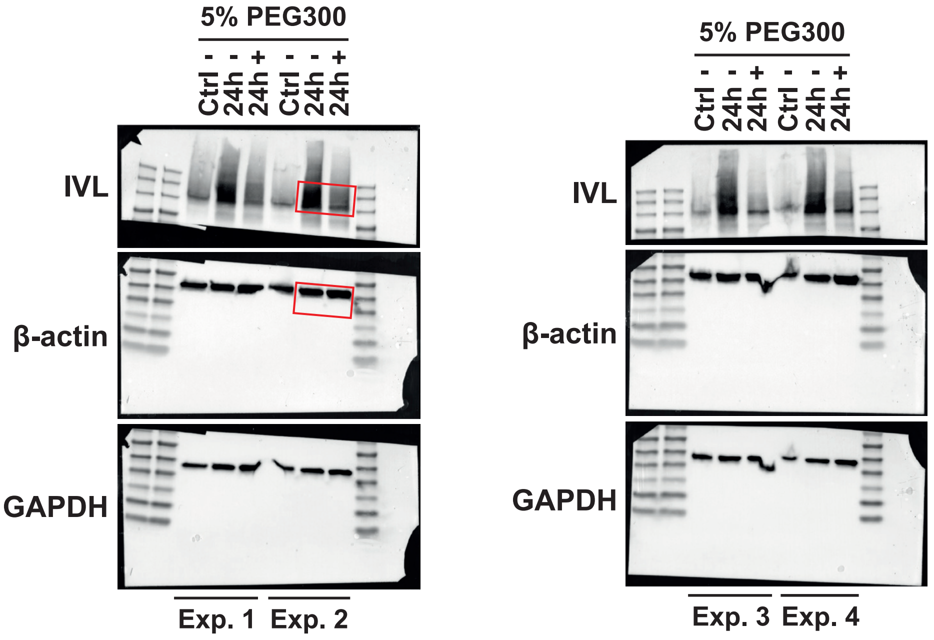

Fig. S4. Western blotting of cells placed in suspension for 24h to induce terminal differentiation. The starting cell populations served as controls (Ctrl). Cells were suspended in the presence or absence of 5% PEG300, then lysed and subjected to western blotting for involucrin (IVL), β-actin or GAPDH (loading controls). Each blot includes colour pre-stained protein ladders (two lanes on left hand side and two lanes on right hand side; Bio-Rad). Lysates from four independent experiments (Exp.) are shown. Red boxes show regions included in Fig. 7E.

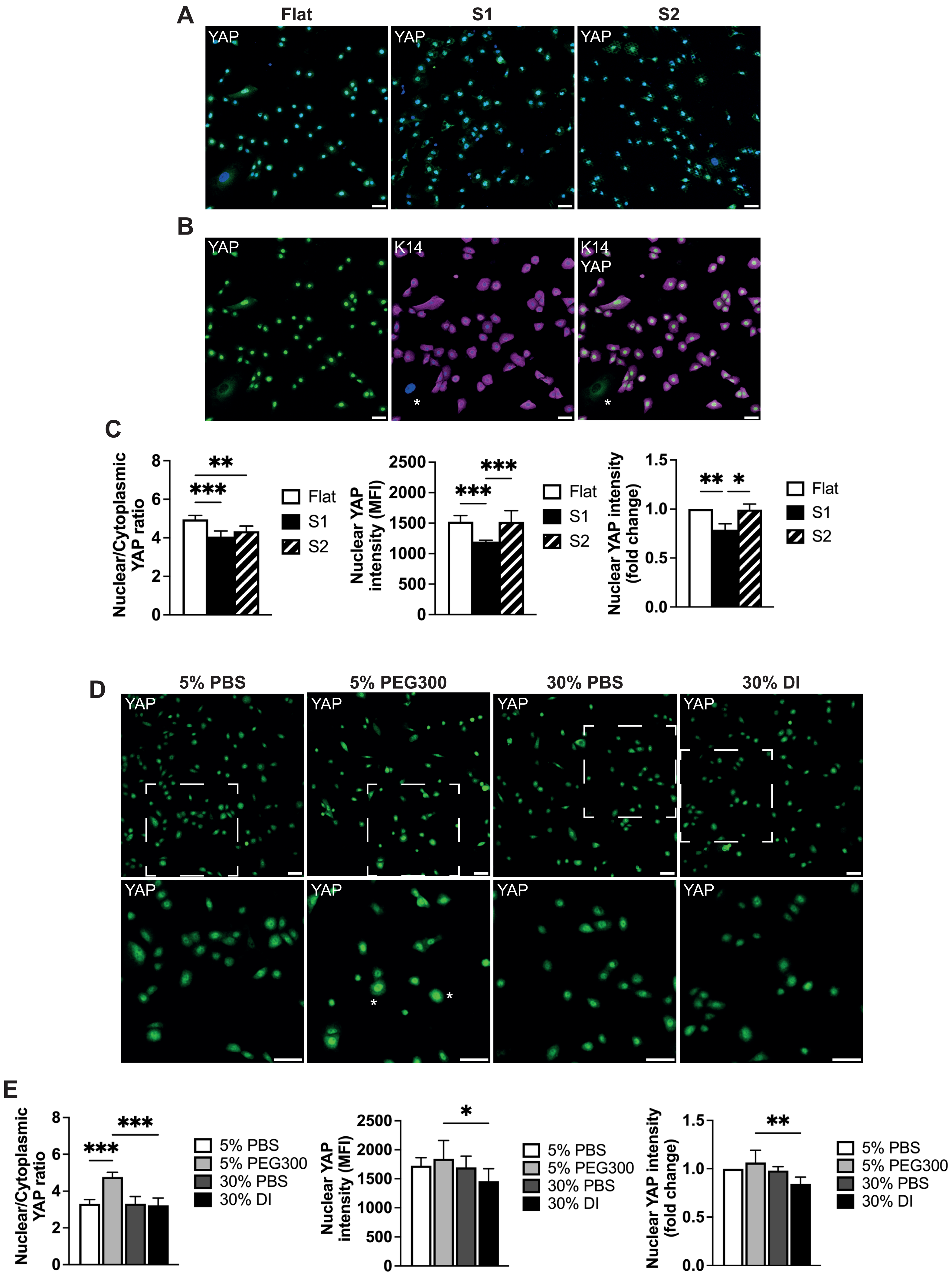
A
Flat
S1
S2
YAP
B
YAP
K14
K14
YAP
C
Nuclear/Cytoplasmic YAP ratio
Flat
S1
S2
Nuclear YAP intensity (MFI)
Nuclear YAP intensity (fold change)
D
5% PBS
5% PEG300
30% PBS
30% DI
E
Nuclear/Cytoplasmic YAP ratio
5% PBS
5% PEG300
30% PBS
30% DI
Nuclear YAP intensity (MFI)
Nuclear YAP intensity (fold change)

Fig. S5. YAP localisation in keratinocytes cultured under different conditions. (A) Representative images of YAP (green) antibody staining of cells grown on flat, S1 and S2 substrates for 12h. (B) YAP (green) and K14 (magenta) staining of cells grown on flat substrate (same field as in A). Asterisk in (B) shows J2-3T3 feeder cell with low nuclear to cytoplasmic YAP ratio. (A, B) DAPI nuclear counterstain (blue). (C) Cells were grown as in (A). Nuclear/cytoplasmic (nuc/cyto) YAP ratio, mean nuclear YAP intensity (MFI) and fold change in mean nuclear YAP intensity. Quantifications are based on >1000 cells per experiment. All results are from 3 independent experiments performed with three technical replicates. Scale bars: 50 µm. Statistics: one-way ANOVA (+ Tukey's) test or Kruskal-Wallis (+ Dunn's) test. Only significant p-values are shown. Error bars: mean + SD. * p-value < 0.05, ** p-value < 0.01, *** p-value < 0.001. (D, E) YAP localisation in cells treated with PEG300 and DI. (D) Representative images of YAP antibody staining (green) in cells grown in the presence of 5% PBS, 5% PEG300, 30% PBS and 30% DI. Cells were cultured for 4h (1h adhesion + 3h incubation with PBS/ PEG300/DI). Boxed areas are shown at higher magnification below. Asterisks: cells with high nuclear YAP after culture in the presence of 5% PEG300. Scale bars: 50 µm. (E) Quantification of the nuclear (Nuc) / cytoplasmic (Cyto) YAP ratio, nuclear YAP intensity and fold change in nuclear YAP intensity (normalised to 5% PBS) of cells grown under the conditions described in (D). Results are from three independent experiments performed with three technical replicates per condition. Results are from >500 cells per experiment. Statistics: one way ANOVA (+ Šidak's) test or Kruskal-Wallis (+ Dunn's) test. Only (near-) significant p-values are shown. Error bars: mean + SD. MFI: mean fluorescence intensity. * p-value < 0.05, ** p-value < 0.01, *** p-value < 0.001.

Journal of Cell Science • Supplementary information

Table S1. Differential gene expression of cells on S1 and S2 at 12h

Ensemble ID	Gene	log2FoldChange	padj
ENSG00000214357	NEURL1B	1 363204441	1 81E-05
ENSG00000138411	HECW2	1 255845964	0 003344149
ENSG00000198959	TGM2	1 253361619	0 000390377
ENSG00000244586	WNT5A-AS1	1 22333588	0 006878916
ENSG00000026025	VIM	1 152684754	3 38E-20
ENSG00000114251	WNT5A	1 13747137	3 98E-13
ENSG00000187498	COL4A1	1 11670945	5 91E-22
ENSG00000120708	TGFBI	1 085029803	4 22E-14
ENSG00000162892	IL24	1 060532447	0 00013212
ENSG00000139211	AMIGO2	0 990445983	4 25E-13
ENSG00000228672	PROB1	0 988811329	0 005637049
ENSG00000143476	DTL	0 954662873	0 002938649
ENSG00000134871	COL4A2	0 92618642	2 95E-11
ENSG00000145040	UCN2	0 885305792	0 008074113
ENSG00000134775	FHOD3	0 881788854	0 000621463
ENSG00000198121	LPAR1	0 84502343	2 23E-05
ENSG00000185989	RASA3	0 836183354	2 26E-05
ENSG00000223749	MIR503HG	0 832643692	0 001904189
ENSG00000111186	WNT5B	0 828474391	0 017525595
ENSG00000135919	SERPINE2	0 816671968	2 39E-12
ENSG00000165434	PGM2L1	0 801206616	0 005242566
ENSG00000188042	ARL4C	0 786894543	6 19E-14
ENSG00000174136	RGMB	0 786232983	3 39E-08
ENSG00000144810	COL8A1	0 780881313	0 000615153
ENSG00000185070	FLRT2	0 778751802	2 23E-05
ENSG00000157168	NRG1	0 774933323	0 005244947
ENSG00000019144	PHLDB1	0 770924159	2 23E-05
ENSG00000143702	CEP170	0 770188835	7 32E-12
ENSG00000115221	ITGB6	0 769290746	3 78E-14
ENSG00000041982	TNC	0 766055716	0 000597229
ENSG00000157350	ST3GAL2	0 758213137	6 83E-07
ENSG00000152894	PTPRK	0 751725333	1 29E-06
ENSG00000053254	FOXN3	0 751555897	3 16E-05
ENSG00000038295	TLL1	0 732940228	1 07E-05
ENSG00000198719	DLL1	0 715862426	0 000469659
ENSG00000166073	GPR176	0 708246258	0 013647874
ENSG00000166825	ANPEP	0 702850422	0 016548469
ENSG00000184916	JAG2	0 699382738	1 46E-11
ENSG00000276043	UHRF1	0 69683001	0 031383832
ENSG00000171992	SYNPO	0 687937058	0 008047698
ENSG00000149557	FEZ1	0 678908019	5 30E-08

ENSG00000124225	PMEPA1	0 673217531	4 44E-09
ENSG00000119326	CTNNAL1	0 672664351	0 002938649
ENSG00000145431	PDGFC	0 672601271	2 14E-05
ENSG00000065308	TRAM2	0 671646148	1 09E-09
ENSG00000151835	SACS	0 665393085	3 29E-06
ENSG00000130635	COL5A1	0 664571171	0 001422002
ENSG00000150687	PRSS23	0 663063734	1 17E-06
ENSG00000271601	LIX1L	0 66146831	0 000542634
ENSG00000137801	THBS1	0 660322415	1 04E-05
ENSG00000152952	PLOD2	0 659106745	0 000463617
ENSG00000012048	BRCA1	0 658364452	0 036675206
ENSG00000152217	SETBP1	0 658300704	1 97E-06
ENSG00000178882	RFLNA	0 656448187	3 74E-05
ENSG00000171310	CHST11	0 648665797	1 77E-11
ENSG00000100234	TIMP3	0 646280356	2 82E-05
ENSG00000131016	AKAP12	0 64611106	0 028967297
ENSG00000165572	KBTBD6	0 644735086	0 001304083
ENSG00000103489	XYLT1	0 641458099	0 002645584
ENSG00000157617	C2CD2	0 629437679	0 000542947
ENSG00000058091	CDK14	0 619014822	1 12E-05
ENSG00000143878	RHOB	0 613675572	0 00049503
ENSG00000138356	AOX1	0 612450848	0 022537481
ENSG00000107957	SH3PXD2A	0 587680218	2 15E-09
ENSG00000173706	HEG1	0 583174618	0 010110281
ENSG00000074590	NUAK1	0 580896651	0 001960941
ENSG00000110031	LPXN	0 568753569	0 029738907
ENSG00000106003	LFNG	0 563191014	0 005885599
ENSG00000154764	WNT7A	0 562482003	4 29E-05
ENSG00000105137	SYDE1	0 561401897	0 009395851
ENSG00000186340	THBS2	0 560381775	0 001904189
ENSG00000113140	SPARC	0 556625885	0 002759414
ENSG00000135723	FHOD1	0 556053441	0 000150818
ENSG00000101384	JAG1	0 555800622	1 29E-05
ENSG00000111266	DUSP16	0 552713995	0 000499577
ENSG00000123094	RASSF8	0 543876039	0 000710332
ENSG00000225697	SLC26A6	0 543114937	0 00097941
ENSG00000147224	PRPS1	0 54179162	0 004052065
ENSG00000103064	SLC7A6	0 540057237	0 005288283
ENSG00000120875	DUSP4	0 531628408	5 30E-08
ENSG00000091409	ITGA6	0 528529385	1 91E-07
ENSG00000108854	SMURF2	0 528011441	4 75E-05
ENSG00000184014	DENND5A	0 522197997	0 000158758
ENSG00000168785	TSPAN5	0 515084891	0 021202771
ENSG00000111012	CYP27B1	0 514314196	0 000140791

Journal of Cell Science • Supplementary information

ENSG00000105875	WDR91	0 514215905	0 005975048
ENSG00000205213	LGR4	0 511723995	0 017238605
ENSG00000136717	BIN1	0 510777009	0 021202771
ENSG00000134369	NAV1	0 510064944	1 81E-05
ENSG00000197461	PDGFA	0 509293398	0 018812628
ENSG00000150630	VEGFC	0 508640552	0 003756972
ENSG00000183688	RFLNB	0 504782706	0 011618324
ENSG00000053747	LAMA3	0 503730027	9 24E-06
ENSG00000106366	SERPINE1	0 502156041	0 004107675
ENSG00000171608	PIK3CD	0 500505544	0 000101924
ENSG00000221926	TRIM16	-0 500658139	0 001936173
ENSG00000081277	PKP1	-0 500719143	0 025965123
ENSG00000178585	CTNNBIP1	-0 502566543	0 017238605
ENSG00000075651	PLD1	-0 50291213	0 003816091
ENSG00000095203	EPB41L4B	-0 503263089	0 02020341
ENSG00000275342	PRAG1	-0 504645423	0 00066807
ENSG00000148832	PAOX	-0 507086241	0 008195909
ENSG00000116962	NID1	-0 507358083	0 001397353
ENSG00000148339	SLC25A25	-0 507389896	0 002297828
ENSG00000010278	CD9	-0 508058812	0 004595172
ENSG00000104413	ESRP1	-0 508599003	0 002275497
ENSG00000148344	PTGES	-0 511457654	0 00135452
ENSG00000187642	PERM1	-0 512748969	0 022375
ENSG00000115616	SLC9A2	-0 518266208	0 005915396
ENSG00000171219	CDC42BPG	-0 51979305	0 029845789
ENSG00000171813	PWWP2B	-0 530650266	0 003708379
ENSG00000167617	CDC42EP5	-0 534788585	0 045063784
ENSG00000144063	MALL	-0 540527861	0 047804101
ENSG00000144452	ABCA12	-0 540546703	0 029062652
ENSG00000073350	LLGL2	-0 541206768	0 00155004
ENSG00000161921	CXCL16	-0 541574373	0 015356816
ENSG00000102349	KLF8	-0 541661869	0 046818374
ENSG00000175893	ZDHHC21	-0 547542939	0 049413094
ENSG00000100439	ABHD4	-0 550139137	0 003348372
ENSG00000176531	PHLDB3	-0 553427062	0 037145078
ENSG00000130202	NECTIN2	-0 555331533	0 000319986
ENSG00000175984	DENND2C	-0 558350384	4 18E-06
ENSG00000197632	SERPINB2	-0 570682798	0 000542634
ENSG00000188112	C6orf132	-0 577403047	0 002027676
ENSG00000146674	IGFBP3	-0 578776655	7 40E-07
ENSG00000136826	KLF4	-0 583564401	8 43E-08
ENSG00000148429	USP6NL	-0 585025296	0 000550816
ENSG00000254470	AP5B1	-0 585970299	2 19E-05
ENSG00000161011	SQSTM1	-0 588384991	2 26E-05

ENSG00000245532	NEAT1	-0 598953671	0 029062652
ENSG00000129757	CDKN1C	-0 60607726	0 007757953
ENSG00000114638	UPK1B	-0 606165121	0 001239945
ENSG00000183111	ARHGEF37	-0 606469658	0 000140791
ENSG00000213020	ZNF611	-0 607975673	0 013089464
ENSG00000167996	FTH1	-0 60815637	0 034143871
ENSG00000041353	RAB27B	-0 616729171	0 047267792
ENSG00000168453	HR	-0 617938542	0 001983963
ENSG00000131737	KRT34	-0 623305253	0 011618324
ENSG00000121552	CSTA	-0 637054992	0 00326711
ENSG00000203837	PNLIPRP3	-0 639011207	0 002407083
ENSG00000156711	MAPK13	-0 640583516	0 011224381
ENSG00000122359	ANXA11	-0 645676963	2 24E-05
ENSG00000170175	CHRNB1	-0 647156029	0 009708673
ENSG00000128422	KRT17	-0 65661002	0 041775248
ENSG00000177054	ZDHHC13	-0 65942415	0 007933074
ENSG00000187091	PLCD1	-0 664228883	0 000112425
ENSG00000254614	AP003068.2	-0 667768855	0 035843038
ENSG00000102897	LYRM1	-0 668881481	3 47E-05
ENSG00000087086	FTL	-0 669268531	0 004387215
ENSG00000143850	PLEKHA6	-0 670104514	0 00707873
ENSG00000109046	WSB1	-0 670344233	4 15E-08
ENSG00000205744	DENND1C	-0 673979152	0 000168814
ENSG00000103067	ESRP2	-0 675574391	0 000539386
ENSG00000179913	B3GNT3	-0 67575629	0 019885004
ENSG00000109654	TRIM2	-0 675904909	0 011842735
ENSG00000167754	KLK5	-0 676127743	0 000641055
ENSG00000125775	SDCBP2	-0 676147662	0 000782475
ENSG00000011422	PLAUR	-0 677204286	1 02E-08
ENSG00000170485	NPAS2	-0 681114439	0 013232101
ENSG00000213853	EMP2	-0 684267683	0 018965754
ENSG00000185262	UBALD2	-0 685754392	0 004724386
ENSG00000133466	C1QTNF6	-0 687122556	2 57E-05
ENSG00000104267	CA2	-0 689819855	0 007964519
ENSG00000173898	SPTBN2	-0 700272013	1 46E-11
ENSG00000095585	BLNK	-0 705283438	0 04078483
ENSG00000133687	TMTC1	-0 705483309	0 001355796
ENSG00000127507	ADGRE2	-0 705916417	0 000635276
ENSG00000163141	BNIPL	-0 707969703	0 012639951
ENSG00000123892	RAB38	-0 709609405	0 028786422
ENSG00000116741	RGS2	-0 718281753	5 47E-05
ENSG00000147676	MAL2	-0 719570574	8 09E-08
ENSG00000137193	PIM1	-0 728786603	5 65E-07
ENSG00000136155	SCEL	-0 729513173	6 68E-19

Journal of Cell Science • Supplementary information

ENSG00000184588	PDE4B	-0 730767548	7 22E-05
ENSG00000130940	CASZ1	-0 733352603	8 83E-07
ENSG00000075213	SEMA3A	-0 734434547	1 76E-06
ENSG00000114166	KAT2B	-0 740982534	0 000116734
ENSG00000125648	SLC25A23	-0 742189192	0 000531291
ENSG00000180914	OXTR	-0 742253369	0 008566296
ENSG00000154227	CERS3	-0 742442424	0 011823534
ENSG00000275234	AC010503.4	-0 742649833	0 002780029
ENSG00000167306	MYO5B	-0 743340637	0 040636586
ENSG00000172183	ISG20	-0 749705099	0 006477966
ENSG00000175130	MARCKSL1	-0 753868399	0 016527353
ENSG00000188215	DCUN1D3	-0 762768602	1 91E-07
ENSG00000167880	EVPL	-0 769781763	0 000143875
ENSG00000184292	TACSTD2	-0 771797768	7 02E-08
ENSG00000117114	ADGRL2	-0 773181584	0 000482346
ENSG00000143375	CGN	-0 774069135	0 001986149
ENSG00000169228	RAB24	-0 774141556	0 001874649
ENSG00000156675	RAB11FIP1	-0 775063186	8 97E-09
ENSG00000196730	DAPK1	-0 783089197	0 022552658
ENSG00000153294	ADGRF4	-0 787725765	1 74E-05
ENSG00000130545	CRB3	-0 788296716	4 83E-07
ENSG00000105855	ITGB8	-0 791951831	1 66E-10
ENSG00000082458	DLG3	-0 796568969	0 000760275
ENSG00000188293	IGFL1	-0 801139965	2 21E-05
ENSG00000223573	TINCR	-0 806145768	1 32E-05
ENSG00000134531	EMP1	-0 815177722	7 34E-06
ENSG00000068137	PLEKHH3	-0 816171019	3 84E-05
ENSG00000184254	ALDH1A3	-0 816226957	0 000647159
ENSG00000087495	PHACTR3	-0 81986883	0 00047486
ENSG00000259583	AC015712.2	-0 822530856	0 000610453
ENSG00000102879	CORO1A	-0 828343945	0 029684737
ENSG00000168970	JMJD7-PLA2G4B	-0 829041498	0 013452563
ENSG00000143590	EFNA3	-0 833739559	0 006477966
ENSG00000143469	SYT14	-0 834573424	0 043348188
ENSG00000042286	AIFM2	-0 834861528	0 009227941
ENSG00000160796	NBEAL2	-0 835755341	5 27E-05
ENSG00000109182	CWH43	-0 841652416	0 035665716
ENSG00000141738	GRB7	-0 843507124	0 002771988
ENSG00000052344	PRSS8	-0 844609897	4 64E-08
ENSG00000168907	PLA2G4F	-0 854038269	0 017962104
ENSG00000162496	DHRS3	-0 859576589	0 000116734
ENSG00000168140	VASN	-0 862180495	0 008042523
ENSG00000111261	MANSC1	-0 862908072	0 040484197
ENSG00000006625	GGCT	-0 863200332	1 76E-05

Journal of Cell Science • Supplementary information

ENSG00000002587	HS3ST1	-0 871566259	2 79E-05
ENSG00000163694	RBM47	-0 886770293	9 22E-09
ENSG00000166535	A2ML1	-0 887737161	1 24E-05
ENSG00000129173	E2F8	-0 892424653	0 044757427
ENSG00000074181	NOTCH3	-0 892831424	0 006678894
ENSG00000076864	RAP1GAP	-0 894176323	0 002943715
ENSG00000136425	CIB2	-0 898575098	0 041395887
ENSG00000176920	FUT2	-0 902941647	0 001239945
ENSG00000197355	UAP1L1	-0 908100225	0 00017317
ENSG00000182010	RTKN2	-0 92750751	6 13E-06
ENSG00000155918	RAET1L	-0 935183116	1 04E-05
ENSG00000167757	KLK11	-0 941362345	0 002944107
ENSG00000261068	AL512274.1	-0 945472004	0 02020341
ENSG00000104808	DHDH	-0 95657253	0 003554113
ENSG00000204632	HLA-G	-0 956943203	0 002519472
ENSG00000163347	CLDN1	-0 965421063	1 84E-07
ENSG00000102243	VGLL1	-0 967665193	1 11E-07
ENSG00000074964	ARHGEF10L	-0 973139228	0 029793282
ENSG00000129353	SLC44A2	-0 980089496	5 91E-22
ENSG00000104140	RHOV	-0 987206561	3 51E-05
ENSG00000140519	RHCG	-1 010560484	0 001354217
ENSG00000170423	KRT78	-1 012387592	0 016471867
ENSG00000131037	EPS8L1	-1 015705029	3 45E-07
ENSG00000175040	CHST2	-1 019689041	5 52E-08
ENSG00000065361	ERBB3	-1 020275805	6 15E-06
ENSG00000005001	PRSS22	-1 024099354	6 07E-08
ENSG00000167656	LY6D	-1 037689799	0 033671094
ENSG00000175505	CLCF1	-1 039955884	0 001362049
ENSG00000176845	METRNL	-1 043820271	5 65E-07
ENSG00000185499	MUC1	-1 046005592	6 07E-10
ENSG00000075673	ATP12A	-1 046694961	0 030574153
ENSG00000175315	CST6	-1 046697245	0 00494806
ENSG00000181885	CLDN7	-1 059110259	5 04E-08
ENSG00000136689	IL1RN	-1 061966764	5 44E-09
ENSG00000127954	STEAP4	-1 063466393	1 25E-08
ENSG00000182580	EPHB3	-1 064412252	6 12E-08
ENSG00000160213	CSTB	-1 065929126	5 65E-07
ENSG00000171346	KRT15	-1 067334694	4 96E-05
ENSG00000167741	GGT6	-1 068536478	0 016471867
ENSG00000179178	TMEM125	-1 08586034	3 45E-05
ENSG00000167759	KLK13	-1 091605802	0 007195782
ENSG00000204385	SLC44A4	-1 100913371	0 042431974
ENSG00000197822	OCLN	-1 103611065	2 29E-09
ENSG00000151715	TMEM45B	-1 108715128	9 02E-06

ENSG00000183742	MACC1	-1 110991461	4 55E-06
ENSG00000197506	SLC28A3	-1 134886398	0 001355533
ENSG00000141527	CARD14	-1 136769302	0 025747686
ENSG00000176092	CRYBG2	-1 140463542	2 30E-06
ENSG00000138316	ADAMTS14	-1 140563143	0 007699827
ENSG00000163472	TMEM79	-1 143963013	0 000197883
ENSG00000178372	CALML5	-1 146759737	0 045838171
ENSG00000186517	ARHGAP30	-1 15317308	0 018965754
ENSG00000188277	C15orf62	-1 154583698	0 001433236
ENSG00000129437	KLK14	-1 155649012	3 29E-05
ENSG00000261040	WFDC21P	-1 156585256	5 55E-05
ENSG00000153292	ADGRF1	-1 16229394	0 001936173
ENSG00000142677	IL22RA1	-1 170821048	1 55E-06
ENSG00000171345	KRT19	-1 202048526	2 23E-08
ENSG00000182175	RGMA	-1 204651194	4 96E-05
ENSG00000136688	IL36G	-1 216704986	5 06E-11
ENSG00000104783	KCNN4	-1 223490317	1 24E-05
ENSG00000102886	GDPD3	-1 234455668	0 009405092
ENSG00000171124	FUT3	-1 235515582	9 69E-11
ENSG00000175121	WFDC5	-1 244906392	0 009433645
ENSG00000163220	S100A9	-1 250740209	0 037857661
ENSG00000137648	TMPRSS4	-1 284147628	1 38E-06
ENSG00000128917	DLL4	-1 294795033	0 048940202
ENSG00000118898	PPL	-1 305640611	4 71E-23
ENSG00000125508	SRMS	-1 319051193	0 011895475
ENSG00000189051	RNF222	-1 323457168	0 001819667
ENSG00000163898	LIPH	-1 328393254	0 001066609
ENSG00000136153	LMO7	-1 331937542	1 36E-41
ENSG00000103034	NDRG4	-1 334298079	0 0026589
ENSG00000189143	CLDN4	-1 339965471	0 019720231
ENSG00000172818	OVOL1	-1 34258794	9 46E-22
ENSG00000141579	ZNF750	-1 352977881	3 78E-14
ENSG00000124215	CDH26	-1 36149672	0 022677546
ENSG00000162069	BICDL2	-1 363281083	5 53E-09
ENSG00000245848	CEBPA	-1 391538784	0 0027576
ENSG00000243708	PLA2G4B	-1 393096849	0 000208705
ENSG00000179477	ALOX12B	-1 394575077	0 012406782
ENSG00000135363	LMO2	-1 40483225	0 045404527
ENSG00000135549	PKIB	-1 420274391	0 000100754
ENSG00000158055	GRHL3	-1 423306765	4 12E-14
ENSG00000183018	SPNS2	-1 429122137	2 79E-09
ENSG00000136542	GALNT5	-1 431402787	5 44E-09
ENSG00000128383	APOBEC3A	-1 448480948	0 027700611
ENSG00000103089	FA2H	-1 486746885	0 000433867

Journal of Cell Science • Supplementary information

ENSG00000092929	UNC13D	-1 496056671	6 08E-10
ENSG00000074317	SNCB	-1 512369175	0 006788476
ENSG00000162572	SCNN1D	-1 518802269	0 04078483
ENSG00000134317	GRHL1	-1 532248082	7 78E-15
ENSG00000143217	NECTIN4	-1 558959398	1 30E-09
ENSG00000166689	PLEKHA7	-1 563904393	4 64E-08
ENSG00000198092	TMPRSS11F	-1 572899696	0 000469659
ENSG00000163216	SPRR2D	-1 579834277	0 005131838
ENSG00000109101	FOXN1	-1 582235019	3 90E-05
ENSG00000203783	PRR9	-1 597197408	0 013370749
ENSG00000186474	KLK12	-1 613099589	0 009403419
ENSG00000237330	RNF223	-1 619340373	3 15E-06
ENSG00000181652	ATG9B	-1 620746385	0 00066807
ENSG00000186212	SOWAHB	-1 692177738	7 88E-05
ENSG00000092295	TGM1	-1 695420654	3 46E-52
ENSG00000188505	NCCRP1	-1 69866081	7 79E-06
ENSG00000127084	FGD3	-1 741210697	0 012815805
ENSG00000188089	PLA2G4E	-1 769220995	7 47E-10
ENSG00000088002	SULT2B1	-1 798157054	0 001940416
ENSG00000167767	KRT80	-1 799462757	5 06E-35
ENSG00000283167	AC140479.7	-1 82132107	0 000237059
ENSG00000105131	EPHX3	-1 826960918	1 12E-08
ENSG00000160145	KALRN	-1 874916533	4 82E-06
ENSG00000172478	C2orf54	-1 879535279	2 46E-13
ENSG00000136695	IL36RN	-1 902550135	2 99E-11
ENSG00000100170	SLC5A1	-1 920423631	0 003221393
ENSG00000203785	SPRR2E	-1 980175269	0 046618723
ENSG00000241794	SPRR2A	-2 004655905	0 001084874
ENSG00000169509	CRCT1	-2 040178093	0 000177324
ENSG00000163435	ELF3	-2 056305456	1 54E-30
ENSG00000152766	ANKRD22	-2 07769143	2 46E-11
ENSG00000130701	RBBP8NL	-2 457699128	3 07E-09

Journal of Cell Science • Supplementary information

Table S2. Antibodies and other reagents used for immunolabelling and Western blots

Antibody/reagent information					Dilution	
**Antibody against:**	**Source**	**Reference**	**Clone**	**Host species**	**Western Blot**	**Immuno-fluorescence**
Involucrin	In-house	Hudson et al., 1992	SY3	Mouse	1:1000	1:1000
Involucrin	In-house	Hudson et al., 1992	SY7	Mouse	1:1000	1:1000
Cleaved Caspase-3	Cell Signaling Technology	9664	Asp175	Rabbit	-	1:500
YAP	Cell Signaling Technology	14074	D8H1X	Rabbit	-	1:100
Keratin-14	Biolegend	906001/906004	Poly9060	Chicken	-	1:1000
HRP-conjugated β-actin	Santa Cruz Biotechnology	sc-47778	C4	Mouse	1:5000	-
Donkey anti-Mouse IgG, Alexa Fluor 594	Invitrogen	A21203	-	-	-	1:1000
Donkey anti-Rabbit IgG, Alexa Fluor 488	Invitrogen	A21206	-	-	-	1:1000
Donkey anti-Rabbit IgG, Alexa Fluor 594	Invitrogen	A21207	-	-	-	1:1000
Donkey anti-Chicken IgY, Alexa Fluor 647	Invitrogen	A78952	-	-	-	1:1000
HRP-conjugated horse anti-mouse IgG	Cell Signaling Technology	7076			1:1000	-
Phalloidin Alexa Fluor 488	Invitrogen	A12379	-	-	-	1:500
DAPI	Invitrogen	D1306	-	-	-	1:1000 (1 μg/ml)

Journal of Cell Science • Supplementary information

Table S3. Oligonucleotide primers used in RT-qPCR reactions.

Table showing the oligonucleotide primers used for RT-qPCR reactions. Primers were designed in Primer3 with the following settings: an amplicon size: 70-110 base pairs (bp), melting temperature: 58-62 °C, G/C content: 40-60% and primer size: 18-22 bp. Where possible primers were designed to cross introns. Only protein coding transcripts were targeted. Primer sequences were validated by confirmed by Ensemble BLASTN.

**Gene:**	**Forward primer:**	**Reverse primer:**
18S	GCAATTATTCCCCATGAACG	GGCCTCACTAAACCATCCAA
GADPH	GAAGAGAGAGACCCTCACTGCTG	ACTGTGAGGAGGGGAGATTCAGT
TBP	GTGACCCAGCATCACTGTTTC	GAGCATCTCCAGCACACTCT
GRHL1	ACTGCCAGATCAAGGTCTTCT	GCTTTGCTTTCGTTCTTCATCC
GRHL3	GTGTTTGACGCGCTCATGTT	CCCATACTTCTCAGAGATCGCA
OVOL1	GTCTCCACGTGCAAGAGGA	GCTGACTGGCACGTAGATCT
PPL	GCAGAGTGACCTGGCTCGGCT	GCCGCATCCGCCTCTAGCAC
TGFBI	TTCACCATCTTCGCCCCTAG	TGACATTGCTGACCAGGGAG
TGM1	GCACCACACAGACGAGTATGA	GGTGATGCGATCAGAGGATTC
VIM	GCTAACCAACGACAAAGCCC	TCCTGCAATTTCTCCCGGAG
WNT5A	TCCTCATGAACCTGCACAACA	CACTTGCAGGCCACATCAG
ZNF750	GCTCACCTTTCCTTCCACCA	GGGATTGTGGGGTGCATGTA
